# Mucoadhesive functionality of cell wall structures from fruits and grains: Electrostatic and polymer network interactions mediated by soluble dietary polysaccharides

**DOI:** 10.1038/s41598-017-16090-1

**Published:** 2017-11-17

**Authors:** Oliver W. Meldrum, Gleb E. Yakubov, Ghanendra Gartaula, Michael A. McGuckin, Michael J. Gidley

**Affiliations:** 1 ARC Centre of Excellence in Plant Cell Walls, The University of Queensland, St Lucia, 4072 Qld Australia; 2 Centre for Nutrition and Food Sciences, Queensland Alliance for Agriculture and Food Innovation, The University of Queensland, St Lucia, 4072 Qld Australia; 3 School of Chemical Engineering, The University of Queensland, St Lucia, 4072 Qld Australia; 4 Chronic Disease Biology and Care Program, Mater Research Institute - The University of Queensland, Translational Research Institute, Woolloongabba, Qld 4102 Australia

## Abstract

We demonstrate the enhancement of intestinal mucin (Muc2) binding to plant cell wall structures from fruit (parenchymal apple tissue) and grain (wheat endosperm) mediated by soluble dietary fibers embedded within cellulose networks. Mucin binding occurs through two distinct mechanisms; for pectin polysaccharides characteristic of fruits and vegetables, it is governed by molecular mucoadhesive interactions, while for neutral polysaccharides, arabinoxylan and *β*-glucan characteristic of cereal grains, the interaction stems from the properties of their polymer network. Based on microrheological and microscopic measurements, we show that neutral dietary fiber polysaccharides do not adhere to intestinal mucin, but are capable of disrupting the mucin network, which facilitates interpenetration of mucin molecules into the polysaccharide mesh. This effect becomes significant in the context of ‘whole foods’, where soluble fibers are incorporated within the gel-like matrix of cellulose-reinforced plant cell wall structures. The result of mucoadhesion assay and analysis of microscopy images points to the critical role of entanglements between mucin and polysaccharides as a lock-in mechanism preventing larger mucin from escaping out of plant cell wall structures. These results provide the first indication that non-pectin soluble dietary fiber may influence mucosal interactions, mucus barrier properties, and transmucosal transport of nutrients.

## Introduction

Plant-based foods are at the core of a healthy human diet, with epidemiological studies highlighting the importance of e.g. whole grain consumption, which significantly reduces the risk of Type II diabetes, colorectal cancer, and stroke^1,2^. Human populations with diets rich in whole-grains, fruits and vegetables experience a considerably lower prevalence of certain cancers compared to those where processed foods and certain types of red meat dominate^3,4^. Starch aside, plant cell walls are the most common structural component in plant-based foods. These complex polysaccharide assemblies represent the main fraction of dietary fiber (DF). Despite the documented significance of DF reported in metabolic and epidemiological studies, the mechanisms that underpin their health promoting properties remain incompletely understood^5,6^. This presents a major challenge from both food technology as well as nutritional and physiological perspectives.

Soluble dietary fiber (SDF) polysaccharides have particular significance for aspects of human health. For example, cereal β-glucan (oat, barley) and pectin (wide range of fruits) provide a cholesterol-lowering effect which in turn reduces the risk of cardiovascular disease^7,8^. From a functional perspective, it is important to consider whether the fiber is isolated in a molecular soluble form or occurs as a part of whole food^9^. In whole foods, the majority of SDFs are embedded within plant cell walls (PCW), where they form complexes with cellulose^10,11^, or deposited within the inter-cellular matrix, in the so-called middle lamella^12^. The presence of food components and the nature of PCW microstructure may have profound effects on how both soluble and insoluble fibers impact digestive physiology^13^.

By definition, SDFs are indigestible by human enzymes in the upper gastrointestinal (GI) tract, but pass through to the colon, largely as intact molecules^9^. However, SDFs are proposed to be able to reduce the rate of enzymatic digestion of e.g. starches and triglycerides, resulting in reductions in circulating glucose and fatty acids, both nutritionally relevant biomarkers^5,14^. During digestion in the upper GI tract, enzymes break down complex food components such as proteins, triglycerides and starch to smaller molecules that can diffuse through the mucus layer and, consequently, get absorbed at the epithelial surface as nutrients. The mucus layer is a viscoelastic fluid, the primary functions of which is to entrap and transport foreign particles and bacteria away from the underlying epithelial surface, as well as to act as a selective diffusion barrier aiding nutrient permeability and absorption^15^. As a secretion, mucus is mainly composed of water (95%) as well as mucin (1–5%), salts, lipids, and secreted protein (0.5–1%)^16^. Mucin is a class of large molecular weight glycoproteins that represent the functional component of the mucus layer. They assemble to form a polymer network, which gives rise to unique viscoelastic properties of mucus. Secreted GI mucins share a common blueprint to their domain structure, with a central region rich in proline, threonine and serine resides (PTS domain) heavily decorated with O-linked oligosaccharides (glycans)^17^. These densely grafted glycans confer mucin its ‘bottle brush’ architecture; many glycans are terminated with negatively charged sialic acid residues giving mucin its hydrophilic nature^18^. The glycosylated domain is flanked by the amino (N-) and carboxyl (C-) termini, which are largely non-glycosylated globular domains featuring von Willebrand assemblies and cysteine-rich globular domains that are unique to secreted polymerizing mucins. These domains are responsible for bonding between mucin monomers through disulfide and hydrogen bonds, as well as hydrophobic interactions, thus enabling assembly of mucins into a 3D network^19^. Although porcine mucin can be readily purified, the majority of preparations (including commercial ones) do not form a gel-like solution upon re-hydration, which is hypothesized to be due to misfolding of mucin terminal domains and distortion of the balance between mucin and non-mucin components within the preparation.

An important consideration for the functionality of SDFs is their mucoadhesive properties, a complex set of adhesive and binding interactions between the material and mucus. Mucoadhesive materials are capable of interacting with mucins via hydrogen bonding, electrostatic interactions and hydrophobic effects; and thus common *in vitro* mucoadhesive polymers represent a wide spectrum of chemistries, including cationic (chitosan), anionic (polyacrylic acid), neutral hydrophilic (poly(ethylene glycol)) and hydrophobic (hydroxypropylmethyl cellulose)^20^. Whilst insoluble fibers (e.g. cellulose) have only limited physiological effect in the upper GI tract, many natural and synthetic soluble polysaccharides have been shown to provide mucoadhesive properties to dramatically change mucus viscosity^21^. Substantial research has been conducted to examine mucoadhesive interactions of individual soluble fibers such as alginate, guar, and pectin^15,21–24^, but no studies reported to date have considered the effect of food-like PCW fiber matrices on the functional component of the mucus layer.

**Figure 1:**
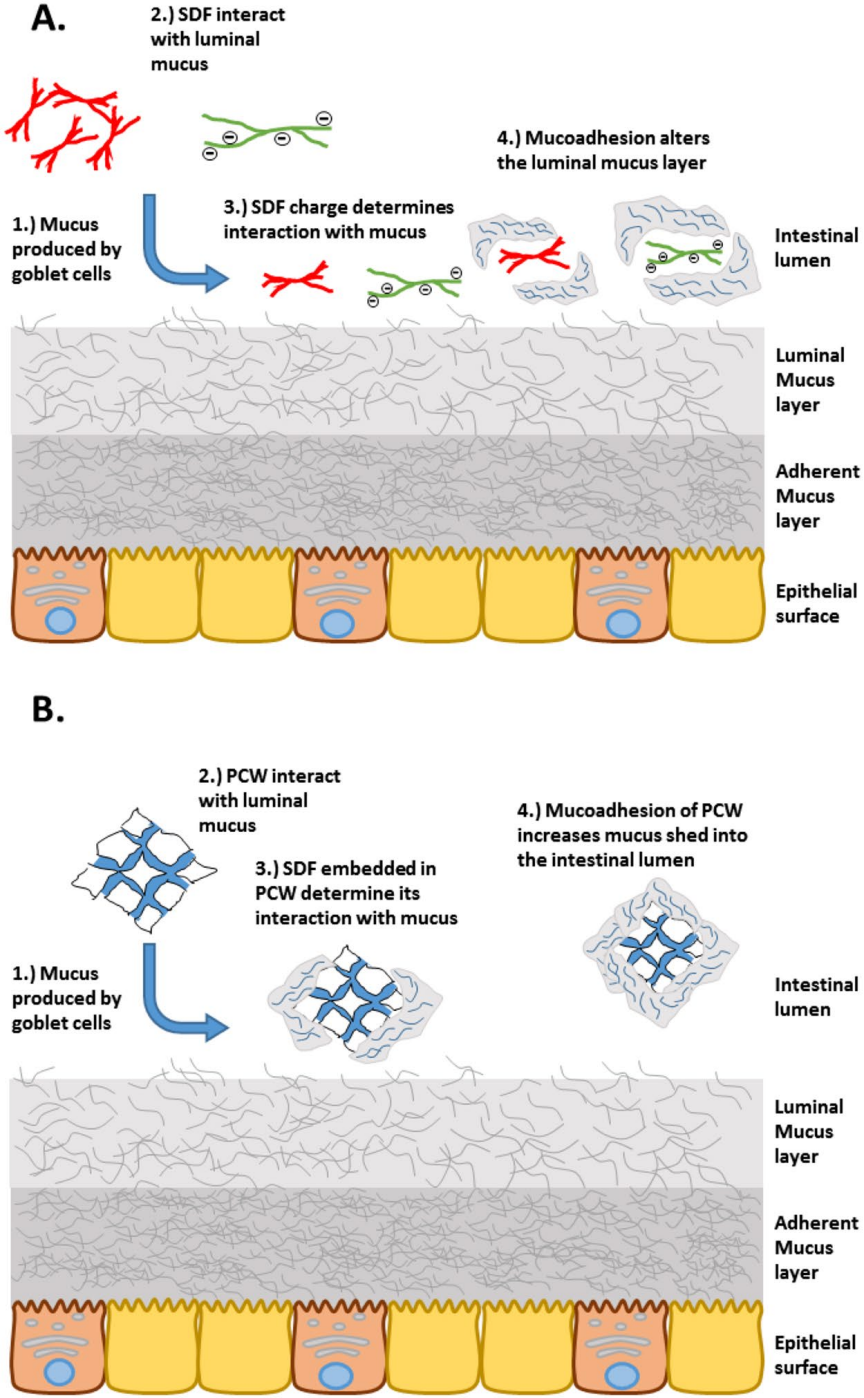
Possible interactions of SDF-containing food particles with the mucus layer. (**A**) Interaction of mucus interfaces with solubilized dietary fiber polysaccharides, which may cause changes in the microstructure within the mucus layer. (**B**) Binding of sloughed-off mucus to plant cell walls within the intestinal lumen.

To define the mucoadhesive functionality of SDFs, we hypothesize (Fig. 1) that SDFs can interact with the mucus layer in a soluble polymer form as well as embedded within the cellulose network of PCW structures. Possible mechanisms include: 1) adhesion of sloughed off mucus to the surface of plant cell walls in the intestinal lumen; 2) interaction of dissolved SDF with the interface of the mucus layer; and 3) changes in mucin microstructure due to SDF penetration/diffusion into the deeper layers of mucus film.

In the present study, we utilized a combination of particle tracking microrheology, confocal laser scanning microscopy (CLSM), and mucoadhesion binding tests to probe the interaction of native preparations of porcine intestinal mucin with soluble polysaccharides of apple pectin, barely *β*-glucan, and wheat arabinoxylan, as well as model food-like PCW structures derived from wheat endosperm and apple parenchyma cells. To the best of our knowledge, this is the first study reporting the interaction of PCW structures with mucus, which is aimed to explore the behavior of whole grain cereals and fruits at the small intestine’s mucus interface.

## Results

### Interaction of isolated SDFs with native intestinal mucins

**Figure 2:**
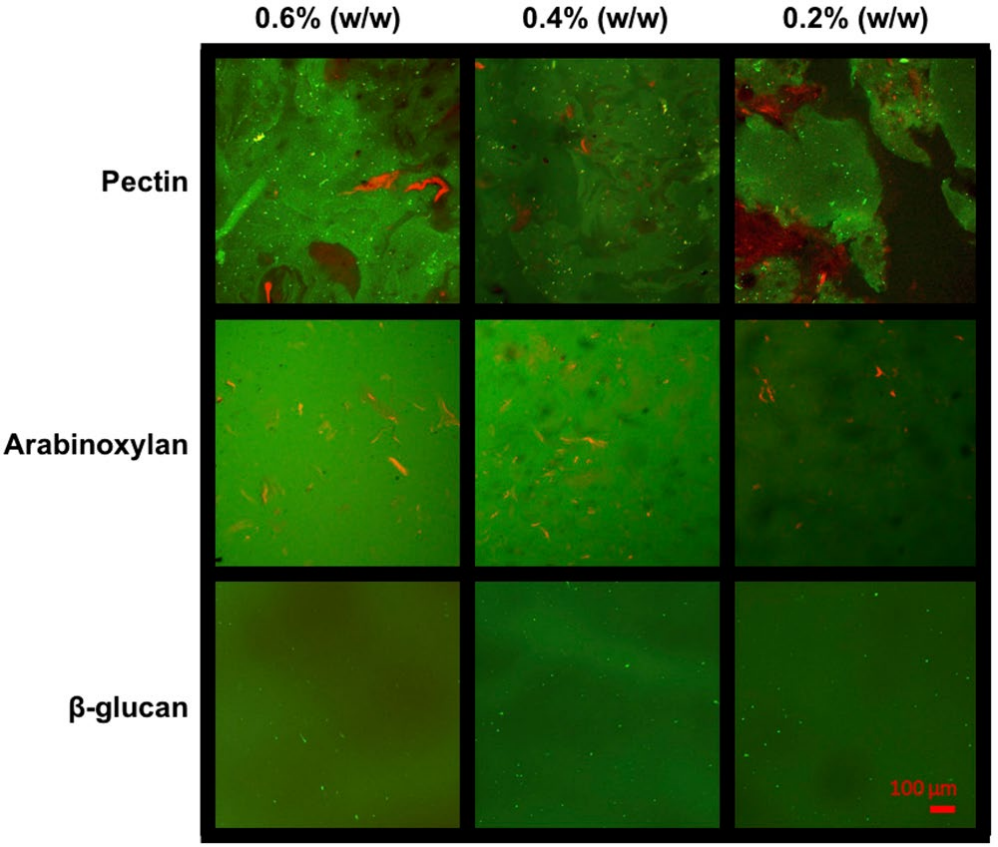
Confocal laser scanning microscopy images of bound 5-DTAF-conjugated soluble dietary fiber (green) with flamingo stained 1% (w/w) mucin (red). The top row shows that mixtures of mucin and pectin form a phase separated solution at lower concentration (0.2% pectin) before a transition into a percolated network at higher concentrations (≥0.4%). Pectin is seen as green aggregates interspersed with red-labelled mucin. In comparison, increasing concentrations of AX and βG show an absence of aggregation compared to that observed with pectin that indicates a lack of interaction between the two polymer solutions. Scale bar 100 μm.

Figure 2 shows representative microstructure of mixtures of purified porcine intestinal mucin (mucin) with increasing concentrations of fluorescent-tagged soluble dietary polysaccharides of pectin, arabinoxylan (AX) and *β*-glucan (*β*G). The images highlight a characteristic interaction between mucin and negatively charged pectin; this result is due to the mucoadhesive nature of pectin which has been reported previously^25,26^ and largely depend on the presence of carboxylic groups that give pectin a net negative charge and hence enable interaction with positively charge amino acids located in the C- and N-termini of mucin^25,26^. At pH 7 in PBS, both pectin and mucin are negatively charged due to the ionization of carboxyl groups in pectin and the sialic acid in mucin with a pKa of about 3–4 and 2.6, respectively^27^. Examining the concentration interaction with mucin (1% w/w) we found that at lower concentrations (0.2% w/w pectin) the mixture appears to phase separate. The phase separation is clearly observed in Fig. 2 (top right panel), which shows the complex multi-phase microstructure of the mucin-pectin mixture featuring mucin-pectin gel-like complexes (green areas), as well as mucin-rich and polymer depleted solvent domains, red and dark areas, respectively. Further addition of pectin (0.4% and 0.6% w/w pectin) results in the formation of a heterogeneous gel-like fluid. We speculate that the density of mucin-pectin complexes increases with pectin concentration, which stabilizes the gel-like structure and increases the water holding capacity of mucin-pectin mixtures^26^. In contrast to pectin, uncharged AX and *β*G appear to show little interaction with mucin, resulting in no observable change in solution microstructure.

**Figure 3:**
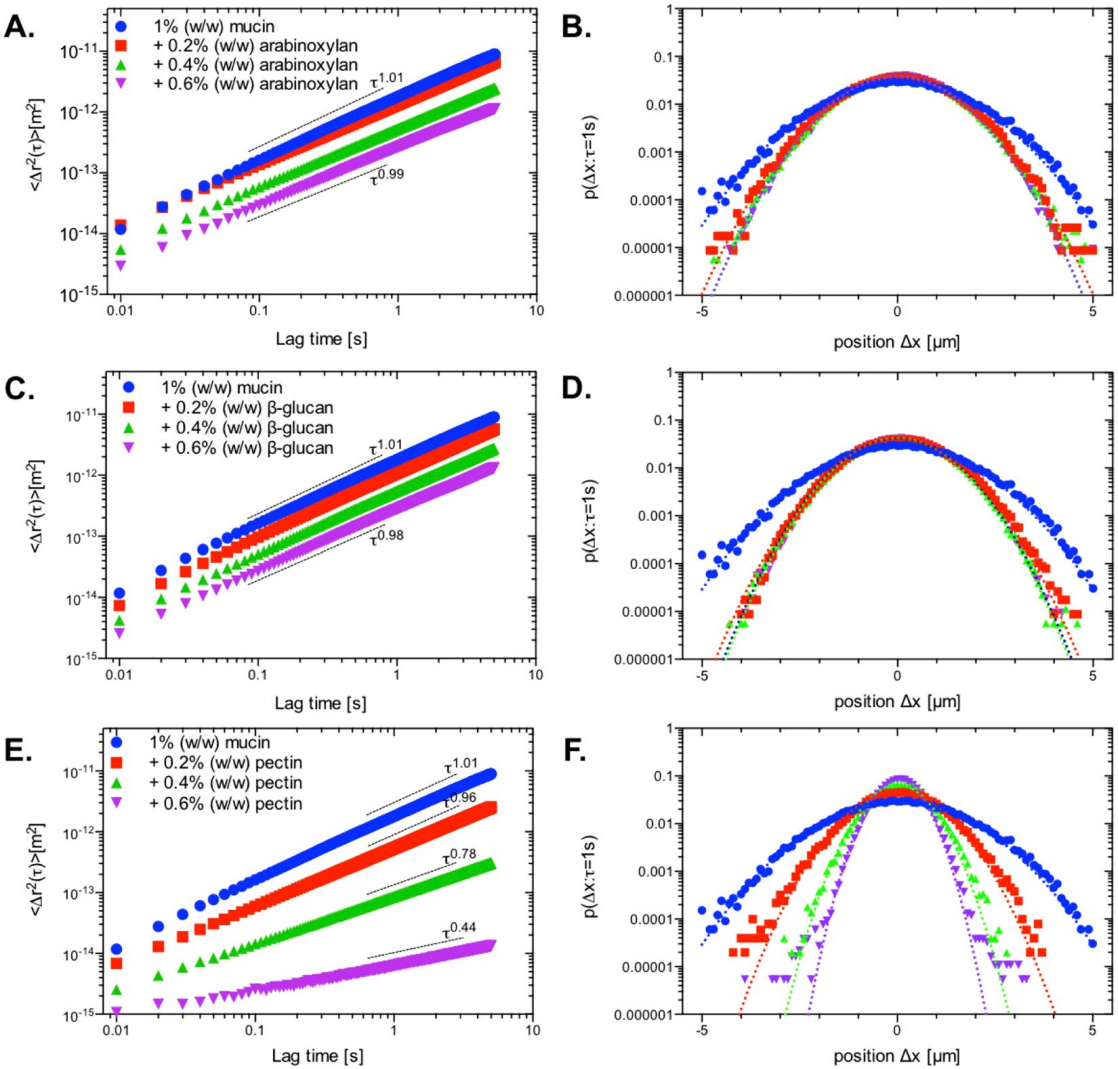
Ensemble averaged MSD curves and van Hove distributions obtained for 1% (w/w) mucin with increasing concentrations of soluble dietary fibers at τ = 1 s. The addition of increasing concentrations of AX or βG (A and C) to 1% (w/w) mucin showed little effect on the ensemble averaged MSD with the corresponding van Hove function following a Gaussian distribution (**B** and **D**). In comparison, pectin (**E**) is shown to induce a gel-like transition with a decrease in the fitted power law scaling of *τ* ^0.96^, *τ* ^0.78^ and *τ* ^0.44^ at 0.2%, 0.4% and 0.6%, respectively. The corresponding van Hove function (**F**) shows that at 0.4% and 0.6%, the values appear to deviate from the Gaussian distribution. Dotted lines represent the Gaussian distributions fitted to the data.

**Figure 4:**
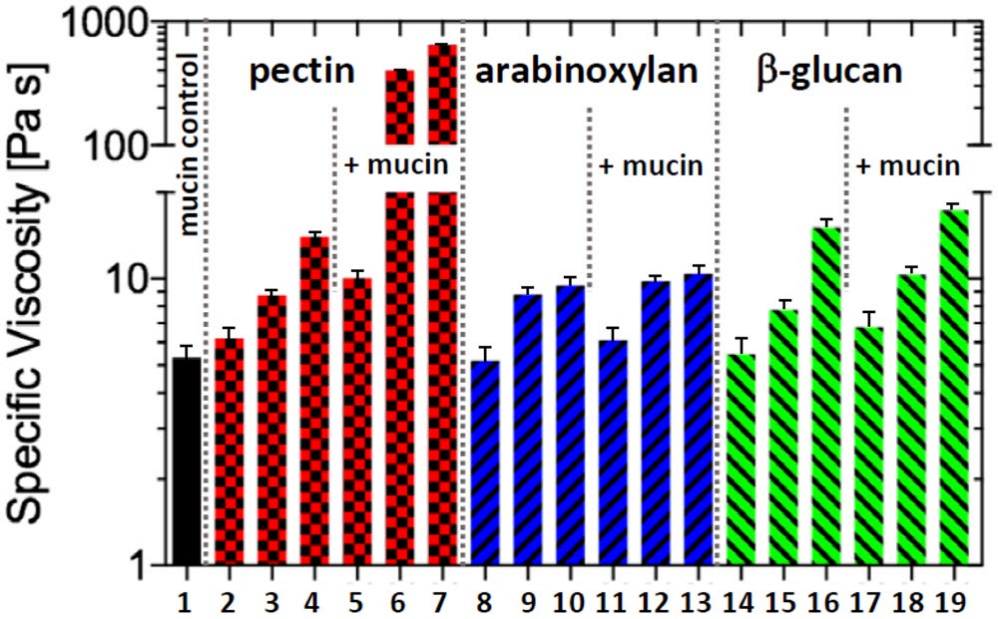
Specific viscosity of 1% (w/w) mucin (**1**), pectin (**2**–0.2% (w/w), **3**–0.4% (w/w), **4**–0.6% (w/w)), AX (**8**–0.2% (w/w), **9**–0.4% (w/w), **10**–0.6% (w/w)), and βG (**14**–0.2% (w/w), **15**–0.4% (w/w), **16**–0.6% (w/w)), as well as mixtures of mucin (1% (w/w)) with soluble dietary fibers at 0.2%, 0.4% and 0.6% fiber concentrations respectively for pectin (**5**–7), AX (**11**–13), and βG (**17**–19). Pectin is shown to dramatically increase the specific viscosity of the solution at higher concentrations (0.4% and 0.6% (w/w)), whereas AX and βG show only a small increase in viscosity.

Despite no observable change in the structure of mucin after the addition of AX or βG, we consider the possibility for these polymers to interact weakly with mucin. In order to probe the mucoadhesive interactions of SDFs further, we have adopted a modified empirical excess viscosity method originally proposed in Hassan and Gallo^28^. The method examines the viscosity (or G”/G’) of the polymer-mucin mixture and compares it to that of individual components, with the difference, called excess viscosity, being reflective of molecular interactions. The viscosity of SDF mixtures with 1% (w/w) mucin solutions were evaluated using passive particle tracking microrheology using 0.5 *μ*m carboxyl-functionalised polystyrene latex particles as diffusive tracers. Representative MSD spectra for each of the mixtures are presented in Fig. 3. The obtained viscosity/complex viscosity values were further converted to the specific viscosity $({\eta }_{{\rm{sp}}}=\frac{\eta -{\eta }_{{\rm{solvent}}}}{{\eta }_{{\rm{solvent}}}})$ as shown in Fig. 4. 1% (w/w) mucin behaved in a purely viscous manner (G′ ≈ 0) with the averaged MSD curves showing a power law fitting of $\langle {\rm{\Delta }}{r}^{2}(\tau )\rangle  \sim {\tau }^{1}$ and a specific viscosity (*η*_*sp*_) of 5.83 mPas. The addition of uncharged polysaccharides such as AX and *β*G resulted in negative excess viscosity, which was particularly evident for AX, whereby viscosity of mixtures is nearly equal to that of pure AX. This suggests that AX and to a lesser extent, *β*G has a disruptive effect on mucin’s semi-dilute network^29,30^, thus rendering the contribution of mucin polymers to the viscosity of the mixture insignificant. For all AX and *β*G concentrations (0.2% w/w to 0.6 w/w) the averaged MSD curves show a power law fitting of $\langle {\rm{\Delta }}{r}^{2}(\tau )\rangle  \sim {\tau }^{0.98-0.99}$, indicating the purely viscous nature of these mixtures. In comparison, the mixtures with pectin show a dramatic change in rheological properties of mucin solutions, which is evident through a marked decrease in power law fitting of MSD spectra which scale with the lag time as *τ* ^0.96^, *τ* ^0.78^, *τ* ^0.44^, for 0.2%, 0.4% and 0.6% (w/w) pectin, respectively (Fig. 3). Already at 0.4% (w/w) pectin, the mixture has appreciable viscoelastic character (tanδ = G′′/G′ ≈ 2.8), with a further increase of pectin concentration resulting in the tanδ decreasing to below 1 (≈0.83). The values of specific viscosity summarized in Fig. 4 shows that excess viscosity for pectin increases dramatically with concentration, for 0.4% and 0.6% (w/w) the excess viscosity is of the order of 100 and 1000, respectively.

To provide further characterization of rheological properties we have examined the particle tracer displacement probabilities, $P({\rm{\Delta }}x=x(t+\tau )-x(t))$, at a lag time of *τ* = 1s. This probability distribution, called the van Hove distribution, shows the variation in the particle travel distance across the ensemble. If each particle experiences the same microrheological environment, the sample is rheologically homogeneous, and the van Hove distribution converges to a Gaussian distribution (*I*_2_(*τ*) = 0). If the ensemble of tracer particles experiences heterogeneous microrheological environments, the van Hove distribution deviates from the Gaussian (Fig. 3 B,D,F dotted line). The extent of this deviation can be quantified by kurtosis, *I*_2_(*τ*), defined in eq. 4; the value of kurtosis is directly proportional to the magnitude of rheological heterogeneity. The van Hove distributions for a range of mixtures of mucin (1% w/w) with AX, *β*G, and pectin are shown in Fig. 3B,D and F, respectively. The corresponding values of kurtosis are summarized in Table 1. For mucin solution as well as for mixtures with AX and *β*G the kurtosis is close to zero, indicating that these solutions are largely rheologically homogenous. We still note, that for pure mucin and its mixtures with 0.6% (w/w) AX and 0.6% (w/w) *β*G, the van Hove distribution functions display a measurable kurtosis that can be clearly distinguished from pure solvent (−0.005 ≤ *I*_2_(*τ*) ≤ 0.005). This observation, while taken with caution, may reflect the same phenomena responsible for the values of excess viscosity of mucin-AX and mucin-*β*G mixtures. The mucin solution, due to its self-associating nature, may display some level of heterogeneity that we detect by analyzing the van Hove distribution. Upon addition of AX or *β*G (c ≤ 0.4% w/w), we hypothesize, the balance of hydrated shells between AX and *β*G and mucin results in a partial dehydration of mucin, which contributes to the disruption of the mucin self-associated network, leading to rheologically homogenous character of the mixed solutions, i.e., *I*_2_(*τ*)~0 ± 0.005. At the 0.6 wt. % concentration of AX and *β*G, *I*_2_(*τ*) increases again, which is expected since heterogeneity typically increases with polymer concentration (Table 1).

**Table 1:** Excess kurtosis of van Hove function, confidence interval 0.01.

	Mucin	Arabinoxylan			***β***-glucan			Pectin		
		+0.2%	+0.4%	+0.6%	+0.2%	+0.4%	+0.6%	+0.2%	+0+0.4%	+0.6%
Excess kurtosis, *I*_2_(*τ*)	0.01	0	0	0.03	0	0	0.02	0.07	0.09	0.12

The van Hove distributions for mucin mixtures with pectin showed a markedly larger kurtosis compared to AX and *β*G mixtures. The values of kurtosis, and hence rheological heterogeneity, are found to positively correlate with pectin concentration. These findings are in excellent agreement with the confocal laser scanning microscopy data, which showed the formation of heterogeneous gel-like structures in mucin-pectin mixtures.

### Interaction of PCWs with native intestinal mucins

The interactions of isolated SDFs showed that pectin is mucoadhesive, while AX and *β*G show muco-disruptive properties. In plant-based foods, however, the majority of SDFs are embedded within a PCW matrix. PCW structures can be considered as micro-gel suspensions which may interact with the mucus layer through surface interactions, such as adsorption and surface wetting. To examine the difference in the interactions of mucins with SDFs embedded within a PCW matrix, we quantified binding of mucin to particulate PCW preparations. In accordance with our conceptual model (Fig. 1), these PCW preparations, unlike isolated and purified SDFs, can mimic more closely the behavior of similar materials found in whole foods. We have used two distinct PCW preparations, apple cell walls which are enriched with pectin and wheat cell walls that are rich in AX and, to a lesser extent, in *β*G^31^. As a negative control, we utilized α-cellulose as a model PCW-derived fiber material devoid of SDFs.

**Figure 5:**
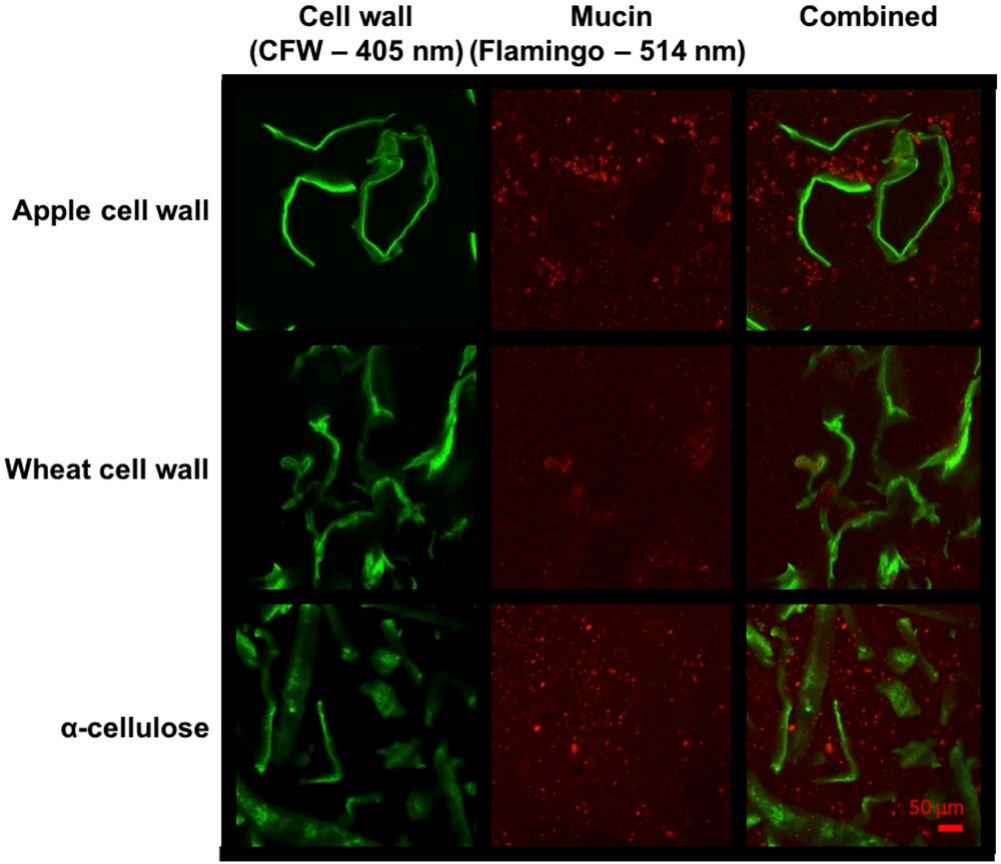
Confocal laser scanning microscopy images of different plant cell wall structures stained with Calcofluor white (green) and flamingo stained porcine intestinal mucin (red). The top row shows the formation of mucin aggregation on the surface of apple cell walls, in contrast to wheat cell walls with little interaction with mucin (middle). The bottom row shows the aggregation of mucus around small fragments of α-cellulose, highlighted by the presence of bright fluorescent particles. Scale bar 50 μm.

**Figure 6:**
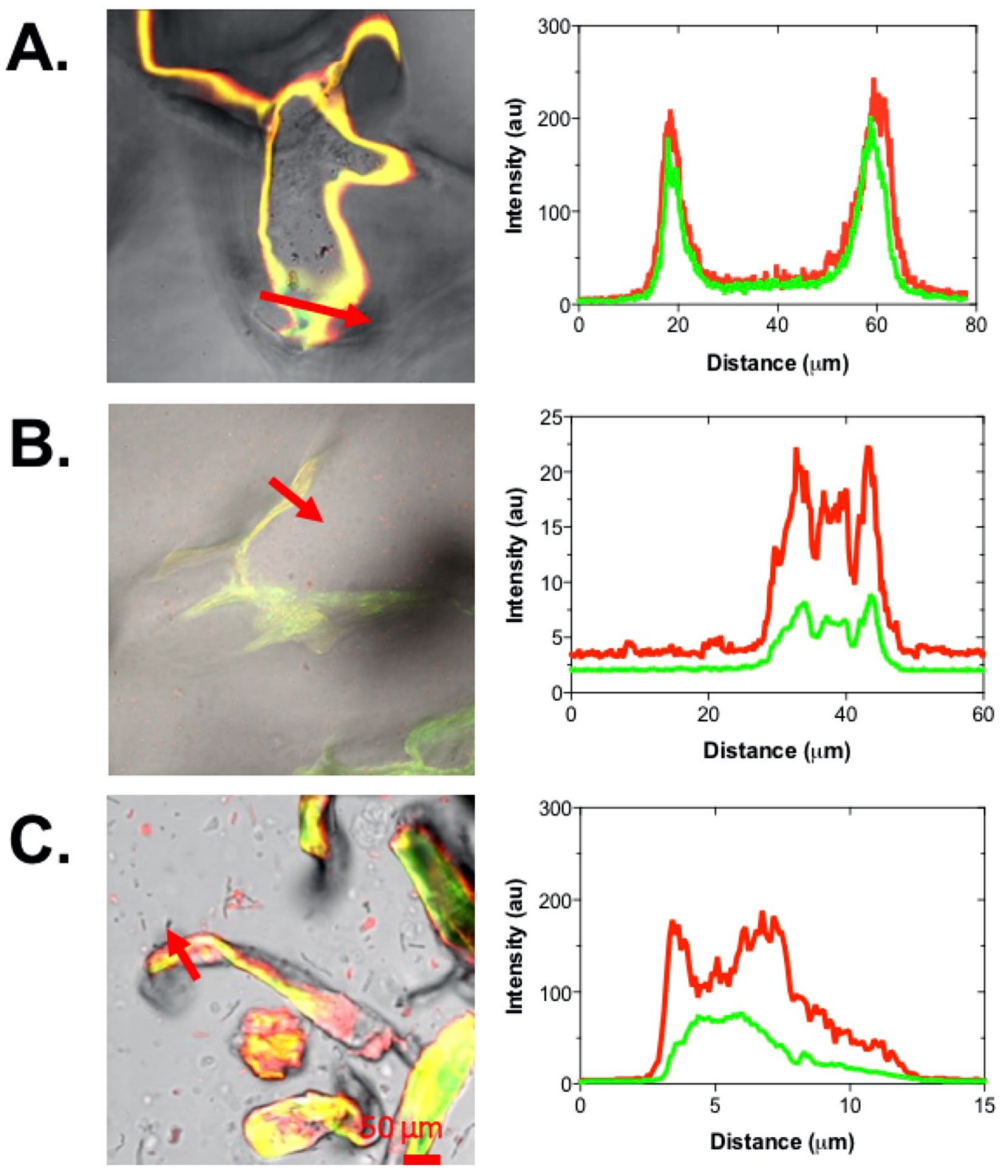
Confocal laser scanning microscopy of cell wall structures from (**A**) apple, (**B**) wheat and (**C**) *α*-cellulose stained with calcofluor white (green) and flamingo-stained mucin (red). The corresponding intensity profiles along the red arrows show mucin (red) accumulated on the surfaces of apple and *α*-cellulose with only a small amount of accumulation on the wheat cell wall. Scale bar 50 *μ*m.

The changes in the microstructure of PCW suspensions upon addition of native mucin were examined using confocal laser scanning microscopy, as shown in Fig. 5. The images of mucin with apple cells walls showed an aggregation of flamingo-stained mucin to the surface of the cell walls as well as the formation of small aggregates around the periphery of PCW particles. As expected, wheat cell walls and α-cellulose suspensions showed less marked changes in the suspension microstructure upon addition of mucin. More detailed analysis of binding to the PCW surfaces showed accumulation of mucin around all types of PCW surfaces as shown by the intensity profiles across particles (marked by arrows in Fig. 6). The fact that mucin binds to surfaces is well-documented; the presence of a wide range of chemistries within the C- and N-termini, as well as within glycosylated domains give rise to the formation of energetically favorable electrostatic and hydrogen bonds, as well as hydrophobic interactions^32–34^. In addition, the strong solvation of mucin makes it energetically favorable to be adsorbed, this process is driven by entropic gains realized via release of the bound water back to the bulk solution upon mucin adsorption^35^.

### *In vitro* mucoadhesive assay: Interaction between PCWs and purified non-denatured porcine intestinal mucin

In order to quantify the binding of mucin to PCW preparations, a mucoadhesion assay was developed to enable comparison of different PCW structures, including preparations treated with hydrolytic enzymes to deplete the SDF content of wall materials. The essence of the method is to utilize the natural propensity of mucins to bind to surfaces via physical binding. Once bound, mucin can be released back into solution using a detergent (in our case anionic surfactant, SDS). The amount of mucin released post SDS treatment is equated to the adsorbed material; the quantification of mucin is performed using the bicinchoninic acid (BCA) microtitre protein assay, which was found to provide sufficient sensitivity for concentrations as low as 25 μg/mL (see Materials and Methods Section for more details). In order to estimate the effective surface area, bovine serum albumin (BSA) was used as a control adsorbate. It should be noted that this method does not provide absolute effective surface area of PCW preparations per unit of mass, but it enables direct comparison between BSA and mucin to inform whether a particular surface is generally protein binding or has more specific mucoadhesive properties. The method also enables a meaningful comparison between PCW preparations and their enzymatically treated counterparts. Enzymatic treatments were used to selectively deplete pectin from apple cell walls and arabinoxylan from wheat cell walls, with the efficacy of the treatments assessed by the monosaccharide analysis presented in Table 2. As seen from Table 2, the enzymatic treatments greatly reduce the relative amounts of galacturonic acid in apple cell walls and arabinose and xylose in wheat cell walls. This selectivity shows that the enzymes successfully reduce the levels of their target polymers.

**Table 2:** Monosaccharide composition of apple (A) and wheat cell walls (B) isolated before and after pectinase and xylanase treatments, respectively.

**A**		
	**Original (%)**	**Pectinase-treatment (%)**
Fucose	0.4	1.1
Rhamnose	2.6	6.0
Arabinose	12.8	24.6
Galactose	4.4	1.3
Glucose	13.1	48.6
Xylose	3.5	7.2
Mannose	0.1	0.4
Galacturonic acid	63.1	10.9
**B**		
	**Original (%)**	**Xylanase-treatment (%)**
Rhamnose	0.5	0.2
Arabinose	22.5	6.5
Xylose	43.7	5.9
Mannose	6.1	23.9
Galactose	0.4	1.8
Glucose	26.8	61.7

**Figure 7:**
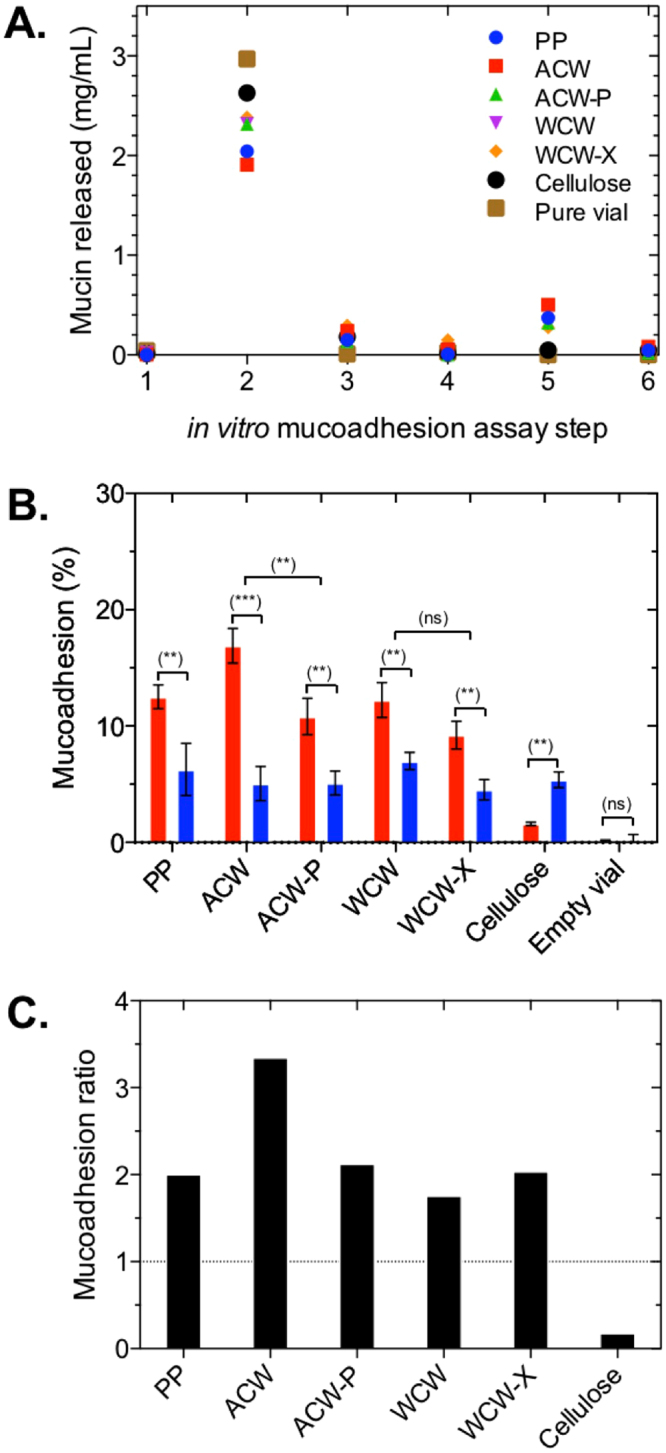
*In vitro* mucoadhesion assay between PCWs and purified porcine intestinal mucin. (**A**) Each step (x axis) represents the amount of mucin released after each washing step. Step 1 – baseline, step 2 – mucin added, step 3 – PBS added, step 4 – PBS added, step 5–10 mM SDS added, step 6 – PBS added. (**B**) Percent mucoadhesion of mucin (red) and BSA (blue) after step 5. Parametric paired t-tests were performed to determine significance. ns = not significant (p > 0.05). ** p value 0.05–0.01. ***p value < 0.01. (**C**) ratio of mucoadhesion normalized by BSA adhesion highlighting the relative binding between mucin and BSA. The notations used: PP - polystyrene particles, ACW - apple cell walls. ACW-P - pectinase-treated apple cell walls, WCW – wheat cell walls, WCW-X xylanase-treated wheat cell walls.

The enzymatic depletion of SDFs is expected to induce a significant change in the surface chemistry of PCW preparations. However, the changes in surface chemistry can be accompanied by the changes in the surface area. Although these changes are possible, we suggest they are small, as BSA adsorption remains essentially unchanged after enzymatic treatment for both apple and wheat PCW preparations (Fig. 7).

In addition to PCW preparations, we have utilized polystyrene particle as positive controls; previous research on the binding of saliva to particles of different surface chemistry and contact angle has established that mucins bind to hydrophilic and hydrophobic surfaces alike, although the amounts bound to hydrophobic surfaces are much larger^36^, making them more suitable as a positive control. We also tested the empty vial to ensure that adsorption to the container was negligible.

The process of this novel mucoadhesive test is presented in Fig. 7A; the 1^st^ column corresponds to the protein detected in pure PCW preparations, which as expected shows negligible values. Upon addition of mucus, the suspensions were shaken to allow mucin to adsorb, and subsequently centrifuged with the supernatant containing unbound mucin material removed. The 2^nd^ column shows the amount of mucin material detected in the supernatant; effectively this amount equals the difference between the added amount of mucin and the bound/entrapped mucin. Next, the PCW precipitates were re-dispersed in the buffer, shaken and further centrifuged to release any entrapped or weakly bound mucin. The 3^rd^ column shows the amount of mucin that has been found in those supernatants, this mucin material corresponds to the entrapped and weakly bound mucin. The analysis of the second wash supernatants is presented in the 4^th^ column, which shows very low levels of protein, indicating that all weakly bound mucin has been liberated from the suspension. The next step was the addition of detergent (SDS) to dislodge strongly adsorbed mucins. As expected the analysis of SDS supernatants, presented in the 6^th^ column, reveals significant amounts of bound mucin across all surfaces except the empty vial, where no protein was detected. As a last step another buffer rinse was performed to illustrate that the levels of remaining mucin are low (see column 6, Fig. 7A).

The amounts of protein (mucin or BSA) released upon the SDS wash are summarized in Fig. 7B and C. Fig. 7B shows the % of bound mucin relative to the initial amount. Fig. 7C shows the same data normalized against adsorption of BSA (i.e. BSA adhesion is set to 1) for each set of particulates. The data, as well as corresponding standard deviations, are summarized in Table 3.

**Table 3:** Mean and SD of mucin and BSA adhered content estimated following treatment with detergent (step 5 in Fig. 7A).

Material	Mucin (%)	SD	BSA (%)	SD	P-value
PP	12.5	1.0	6.3	2.2	0.0113
ACW	16.9	1.5	5.1	1.5	0.0006
ACW-P	10.8	1.6	5.1	1.0	0.0065
WCW	12.2	1.5	7.0	0.7	0.0055
WCW-X	9.2	1.2	4.5	0.9	0.0056
Cellulose	1.6	0.1	5.4	0.7	0.0021
Control	~0	0.2	~0	0.7	1.0

The notations used: PP - polystyrene particles, ACW - apple cell walls. ACW-P – pectinase-treated apple cell walls, WCW – wheat cell walls, WCW-X – xylanase-treated wheat cell walls. All experiments were performed in triplicate.

Fig. 7A and C shows a number of important trends with statistically significant (p < 0.05) enhancement of mucin binding compared to BSA with the exception of α-cellulose. The difference is particularly significant (p < 0.01) for ACW preparations, which show a 3-fold increase in mucin binding compared to BSA (Fig. 7B). The fact that this trend is reversed for α-cellulose suggests that hydrophobic surfaces (PP) and hemicellulose-containing PCW surfaces have selective affinity towards mucin, rather than any generic property of mucin preparation that makes it adsorb stronger compared to BSA. We show BSA binds to hydrophobic polystyrene particles and to an equal degree to plant cell walls and cellulose particles. This strongly indicates the mechanism is governed by hydrophobic interactions. For the polystyrene particles the contact angles have been measured previously^36,37^ while for cellulose material the hydrophobic nature of (100) and (200) facets of cellulosic micro-fibrils have recently been modelled by Oehme, *et al*.^38^.

The treatment of ACW with pectinase (ACW-P) and partial removal of pectin, resulted in a reduced level of mucin binding, with no changes to BSA binding, clearly showing that pectin is instrumental for the mucoadhesive properties of ACW. We note that pectinase treatment does not entirely remove pectin as shown in Table 2a, and hence mucoadhesion, albeit reduced for the ACW-P preparation, is attributed to the presence of residual pectin. Surprisingly, WCW showed mucoadhesion on par with PP and ACW-P, despite AX and *β*G being non-mucoadhesive in a purified form. Upon treatment with xylanase, a measurable decrease in mucin binding was observed, but the change is small relative to pectinase treatment of ACW indicating that the presence of *β*G, a SDF not directly affected by xylanase treatment, may provide an additional contribution to the mucoadhesive character of WCW. The remarkable mucoadhesive character of WCW shows the presence of SDFs within the cellulose structures of PCW has a major effect on mucin binding compared to isolated SDF polysaccharides.

Based on the rheological, microscopic and mucoadhesion results, we propose that the disruptive interactions of AX and *β*G with mucin have a crucial effect on mucoadhesion. While molecular interactions are a key driver of the mucoadhesion process with pectin, with AX and *β*G the interactions may stem from their polymer network properties. In a similar way to SDF-mucin polymer mixtures, mucin and isolated AX or *β*G form a polymer mixture, whereby mucin chains are retained within the polysaccharide mesh of the neutral SDFs. In the case of PCW preparations, similar to its interaction with polymer solutions, mucin can penetrate into the AX/*β*G mixed gel which by itself is confined within the cellulose structure, and hence become effectively immobile. Thus, instead of direct molecular interactions between the SDFs and mucins, we encountered a different type of interaction enhanced by weak interactions allowing for mucins to interpenetrate into the SDF gel as clearly shown in the CLSM images of WCW with added mucin (Fig. 5). This entrapment was strong enough to be stable to copious buffer washes, and only disrupted after the addition of a detergent, thus providing the basis for mucoadhesive properties of WCW.

## Discussion

In this study we tested the hypothesis that purified SDFs isolated from fruits (pectin) and grains (AX, *β*G) interact differently with mucin compared to PCW preparations from fruits and grains. We showed, pectin has qualitatively different interactions with mucin compared to AX and *β*G. In-line with earlier reports, pectin binds with mucin to show a typical mucoadhesive functionality^22^. This interaction translates to the mucoadhesive behavior of pectin-rich plant cell wall structures that show marked mucoadhesive capacity, which can be reduced by partial removal of pectin using pectinase treatment.

**Figure 8:**
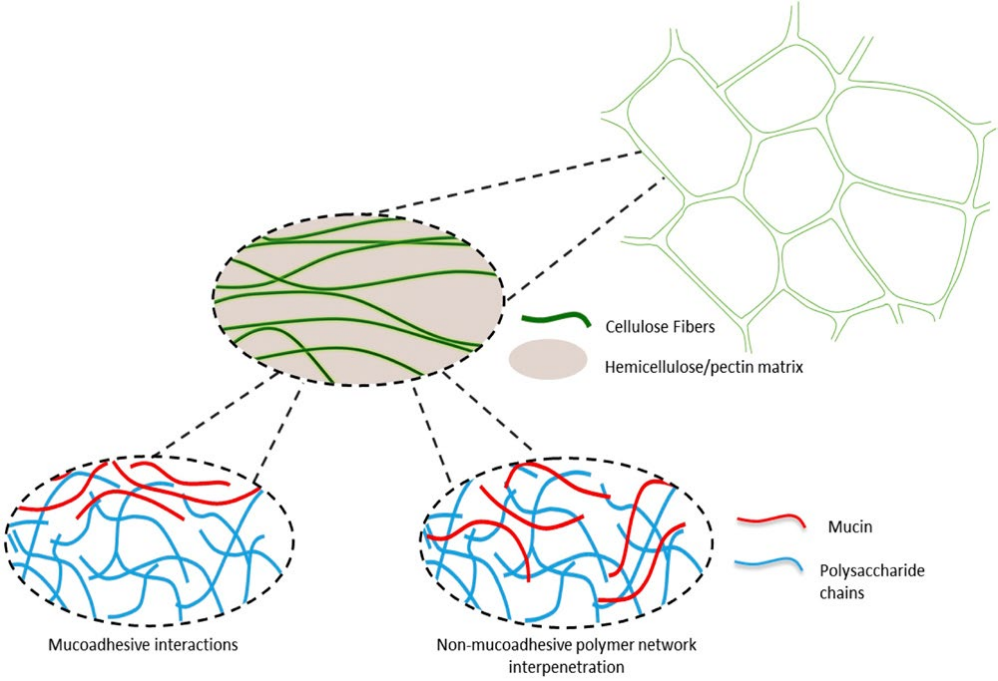
Proposed mechanism of interaction of PCW components with intestinal mucins.

Surprisingly, we found AX and *β*G containing wheat cell walls also show significant mucoadhesive functionality. Despite a lack of binding in an isolated form, the SDFs appear to be instrumental for enhancing mucoadhesive functionality of plant cell walls. Based on the microscopy data shown in Figs 5 and 6, we propose that mucin molecules, when unhindered by strong adhesive interactions, can penetrate or blend into SDF-rich gel-like domains at the periphery of wheat cell walls structures, displaying mucoadhesive-like functionality. The different mechanisms proposed for mucoadhesion of apple and wheat cell walls are schematically illustrated in Fig. 8.

In the context of this hypothesis, there is a potential role of weak interactions between AX/βG and mucins to impact how AX- and βG-rich foods interact with intestinal mucus and, in particular, interfere with the mucus layer at the interface of the intestinal lumen to influence mucus barrier properties. Understanding the mechanisms that govern the interaction of whole PCWs and soluble dietary fibers with the mucus layer may have important implications for the digestion and metabolism of nutrients related to human health. Mackie, *et al*.^39^ previously showed the addition of 10% oat bran (containing AX and *β*G) to the diet had no significant effect on protein or starch digestion while significantly decreasing triglyceride absorption in the diet. Furthermore, they showed a ~60% decrease in the diffusivity of 100 nm particles from oat bran fed mucus. While Gunness, *et al*.^40^ showed a 50% reduction in bile acid active transport across *ex vivo* ileum tissue after 40 minutes (P < 0.001) and a 32% decrease in jejunal microvilli heights of pigs fed diets containing 7% oat *β*G. The current study has shown that there are previously unreported interactions between cereal SDFs or PCWs that could underlie these *in vivo* observations. In addition, this study has highlighted differences in mucin interactions between fruit-based and cereal-based PCWs and SDFs. Both fruits and wholegrain cereals are important components of a healthy diet: the difference in the ways in which their PCWs and SDFs interact with mucin may be a part of the reason why each is important to a healthy diet.

Finally, in the course of this work, we have developed a mucoadhesion assay that enables quantification of bound mucin to the particle surface of various materials. This method is shown to be suitable to compare various plant cell wall preparations and the effect of enzymatic modifications on mucoadhesion.

## Materials and Methods

### Ethics statement and reagents

All animal procedures were approved by the University of Queensland Animal Ethics Committee (ANRFA/QAAFI/424/14). All experiments were performed in accordance with the approved procedures. Materials except otherwise specified were sourced from Sigma-Aldrich (St. Louis, Missouri, USA) and used as received. All solutions were prepared using deionized and filtered water (filter pore size: 0.22 μm, resistivity of 18.2 MΩ*cm, Sartorius Stedim Biotechnology). α-cellulose and 0.5 *μ*m polystyrene particles were exhaustively dialysed against deionised H_2_O prior to use.

### Isolation and identification of porcine intestinal mucin

Porcine intestinal mucin was used as a model for human mucin. Fresh porcine intestines were collected immediately after euthanasia, cut into sections (ca. 60 cm long) and inverted to expose the internal mucosal surface. The internal surface was briefly rinsed with ice-cold PBS before the loose mucus layer was gently removed by passing each section between two glass rods. Mucus scrapings were pooled and mucins were extracted under non-denaturing conditions in a 50:50 ratio of extraction buffer containing 0.1 M NaCl, 0.04% sodium azide, as well as 0.1 M EDTA, 1 M aminohexanoic acid, 0.05 M benzamidine and protease inhibitors for 68–72 hours (4 °C)^41^. Mucins were isolated from non-mucin components using two rounds of CsCl isopycnic density gradient centrifugation in a Beckman L-100 ultracentrifuge (Beckman Ti 45, 72 hours, 40,000 rpm, 12 °C). After the first round (starting density 1.4 g/mL), 20 fractions of increasing density were analyzed for UV absorbance (280 nm), density, and their glycoprotein content using periodic acid/Schiff’s staining (PAS) (Supplementary Information 1). Mucin-rich fractions identified by dot-blot analysis using PAS staining were pooled and following a second round of CsCl isopycnic density gradient centrifugation (starting density 1.5 g/mL). Mucin-rich fractions identified using dot-blot analysis were pooled and exhaustively dialyzed against deionized H_2_O and lyophilized. For analysis, mucin was gently re-suspended in 10 mM phosphate buffer (pH 7) and allowed to rehydrate for at least 48 hours (4 °C). Muc2 mucin was confirmed as the dominant glycoprotein using mass spectroscopy (Supplementary Information 2). Briefly, samples were digested with trypsin, reduced and alkylated before digested peptides were separated from glycosylated domains and salts using a C-18 Zip-Tip (Merck Millipore). Samples were analyzed by LC-MS/MS using a TripleTOF 5600 (ABSciex) with a Nanospray III interface. The MS/MS analysis showed Muc2 to be the most abundant glycoprotein using the SwissProt database (version using the MASCOT search engine via the Australian Proteomics Computational Facility)^42^.

### Isolation of apple parenchymal tissue

Fresh ripe apples (Fuji) were purchased from a local store in St. Lucia, Brisbane (Australia) with parenchymal tissue isolated using a phenol-buffer extraction method^43^. Briefly, fresh tissue (approximately 100 g) was suspended in chilled buffer (1.2 mM CaCl_2_, 2.0 mM MgCl_2_, 0.5 mg/mL KCl, 60 *μ*g/mL ascorbic acid, 4 mg/mL malic acid, and 1 mg/mL sodium sulfite, pH 3.5, adjusted with 5 M NaOH) with 3 mM Triton X-100 and blended for 30 seconds in a blender. The detergent was washed off with chilled buffer (4 °C) using a Buchner funnel with a 0.45 *μ*M PTFE membrane under vacuum until foam disappeared. The ACW preparation was then suspended in chilled acetone: water (60:40, v/v), transferred to a Buchner funnel and washed with 4 volumes of acetone: water. The sample was then solvent exchanged with three volumes of ethanol: water (70:30, v/v) before a second wash with equal parts ethanol and acetone (50:50, v/v) and dried overnight under vacuum. Monosaccharide analysis was determined from individual sugar content on the basis of dry weight following the method of Pettolino, *et al*.^44^. Samples were analyzed using GC-MS using a high polarity BPX70 column.

### Isolation of wheat endosperm tissue

Wheat endosperm cell walls were isolated from the starchy endosperm at a digestion temperature of 37 °C^31^. Briefly, the white starchy endosperm was removed from the endogenous enzyme-inactivated grain (Lincoln Vari.) using a ‘popping’ technique to physically separate the tissue layers. The isolated endosperm was incubated in PBS buffer containing alpha amylase, protease and amyloglucosidase at 37 °C (48 hours) to remove starch, proteins and oligosaccharides. The extent of starch and protein hydrolysis was confirmed with iodine solution and light microscopy. After hydrolysis, the mixture was filtered using a 20 *μ*m screen under running water with isolated cell walls freeze dried for subsequent analysis. Monosaccharide composition was analyzed following the alditol acetate method^44^ using 5 mg of sample that was hydrolyzed with 72% sulfuric acid, reduced with sodium borodeuteride in dimethyl sulfoxide (DMSO) and acetylated using 1-methylimidazole followed by acetic anhydride. The alditol acetate was then extracted into dichloromethane and analyzed using GC-MS (QP2010 Ultra, Shimadzu, Japan) using a high polarity BPX70 column with myo-inositol used as an internal standard.

### Fluorescent conjugation of soluble dietary fiber

Commercially available soluble apple pectin (classic CU-L 051/13, lot 01307706, Herbstreith & Fox KG, Pektin-Fabrik Neuenbürg, Germany), *β*-glucan (medium viscosity barley, lot 90802, Megazyme, Bray, Ireland) and arabinoxylan (medium viscosity wheat, lot 40302a, Megazyme, Bray, Ireland) have been previously analyzed within our research group and have been shown to function as a mimic for creating plant cell wall models^45–47^. Soluble polysaccharides were fluorescently labelled with 5-(4,6-dichlorotriazinyl) aminofluorescein (5-DTAF) reactive dye. Briefly, 0.2 mM 5-DTAF was added to polysaccharide solutions in deionized water (10 mg/mL) and allowed to dissolve at room temperature before 10 mM Na_2_SO_4_ was added slowly over 2 minutes. To initiate the reaction, pH was raised to 10 using 10% (w/v) NaOH and monitored at room temperature. After two hours, the reaction was quenched by the addition of two volumes of ethanol/sodium acetate buffer mixture (1:2 ratio of 0.055 M sodium acetate (pH 5.4): ethanol). Unbound 5-DTAF was washed with sodium acetate: ethanol solution using a Buchner funnel with a 0.45 *μ*M PTFE membrane under vacuum until the filtrate appeared clear. Fluorescently-labelled polysaccharides were then progressively dehydrated with increasing concentrations of ethanol before a final wash with acetone and dried under vacuum overnight in the dark.

### Enzymatic treatment

*Endo*−1,4-*β*-xylanase solution was prepared by mixing 200 *μ*L of *endo*−1,4-*β*-xylanase M6 (rumen microorganism, Megazyme, Bray, County Wicklow, Ireland) in 100 mL of 100 mM sodium phosphate buffer (pH 6). Pectinase solution was prepared by mixing 200 *μ*L of pectinase (*Aspergillus aculeatus*, Sigma Aldrich) in 100 mL of 0.1 M sodium phosphate buffer (pH 6). These enzyme concentrations were selected based on the activity of each enzyme and the amount of substrate present in each preparation. Apple and wheat cell wall preparations were added to 10 mL of enzyme solution overnight at room temperature. After incubation, cell wall preparations were washed three times in deionized water (10 minutes, 10,000 g) and stored in 0.04% sodium azide solution for further analysis.

### *In vitro* mucoadhesion assay of PCW particulate preparations

Plant cell wall mucoadhesion was analyzed *in vitro* in accordance with a modified protocol originally reported by Aguilar-Rosas, *et al*.^48^. A volume of native porcine intestinal mucin (3 mg/mL) and bovine serum albumin (BSA, Sigma Aldrich, 3 mg/mL) were separately added to the suspension of either apple, wheat or *α*-cellulose materials (10 mg/mL) and agitated for 20 minutes at room temperature in 10 mM pH 7 phosphate buffer. The mucin- and BSA-material suspensions were then centrifuged (20,000 g, 20 minutes) before the supernatant containing unbound mucin or BSA was removed. The solutions were then washed two further times with a PBS buffer solution before a single wash with a detergent (10 mM SDS) to remove the bound mucin and BSA. This was followed by a final wash with PBS as a control. The amount of mucin and BSA present in the supernatant after each step was determined using a bicinchoninic acid (BCA) microtitre protein assay. Briefly, 2 mL of BCA working-reagent (50 parts reagent ‘A’ containing BCA detection reagent in a 0.1 M sodium hydroxide to 1 part reagent ‘B’ containing copper sulfate) was added to the supernatant. After incubation for 2 hours at 37 °C, the absorbance was measured at 562 nm (Thermo Fisher Scientific). The amount of mucin and BSA absorbed to the surface was evaluated by analyzing the material that was released after SDS treatment. Additionally, the amount of bound and trapped material was estimated by the difference between the amount of added mucins and that found in the supernatant of the PCW suspension. The analysis was performed in triplicate with the amount quantified using a standard curve for mucin (concentration range 25–2000 *μ*g/mL; R^2^ = 0.996, at pH 7) and BSA (concentration range 25–2000 *μ*g/mL; R^2^ = 0.999, at pH 7). Control samples were performed using an empty vial in order to discard any possible interaction between the container material and mucin or BSA.

### Particle tracking microrheology

For particle tracking experiments, 0.5 *μ*m fluorescent carboxyl-activated polystyrene microspheres (1:2500 dilution from 2.6% stock, Polysciences Inc., Warminster, Pennsylvania, USA) were embedded in mucin-SDF solutions buffered with 10 mM pH 7 phosphate buffer. Plane non-concave microscope slides were used with secure-seal spacers (Grace Bio-Labs, Oregon, USA) between the slide and coverslip. Solutions were allowed to equilibrate for over 24 hours before particle trajectories were recorded using a Phantom × 7.3 fast-action camera coupled to an inverted Nikon Eclipse Ti fluorescent microscope (green CoolLED light source) with a 100x oil immersion objective. Videos were recorded at 100 frames per second for a total of 1500 frames at a resolution of 1152 × 1152 pixels. 20 videos were recorded per sample at random locations that were at least 20 *μ*m from the sample edge. At least 4000 individual trajectories were recorded for each sample. The particle positions and the MSD were calculated using a MATLAB (version 2016a) routine that used a modified version of the algorithm developed by Crocker and Grier^49^. The mean square displacement (MSD, $\langle {\rm{\Delta }}{r}^{2}(\tau )\rangle$), was then determined from the trajectory of each particle as follows:

1 $$MSD(\tau )=\langle {\rm{\Delta }}{r}^{2}(\tau )\rangle =\,\langle {(\mathop{r}\limits^{\rightharpoonup }(t+\tau )-\mathop{r}\limits^{\rightharpoonup }(t))}^{2}\rangle $$

where, $\mathop{r}\limits^{\rightharpoonup }(t)$ and $\mathop{r}\limits^{\rightharpoonup }(t+\tau )$ represents the initial and final position of the centre of mass of the pixel at each time lag, *τ* Each experiment was repeated in triplicate with the stochastic motion averaged over many particle tracks and used to interpret the microenvironment of the tracked particles. Particle diffusion was described as:

2 $$\langle {\rm{\Delta }}{r}^{2}(\tau )\rangle =\,2nD{\rm{\Delta }}{t}^{a}$$

where *n* = 2 applies to the two-dimensional trajectories analysed, *D* represents the diffusion coefficient, and Δ*t* represents the given time interval. For a sphere suspended in a medium, the exponent (*a*) of the power law fit to the *MSD*(*τ*) is different for diffusion from a viscous (*a* = 1) and a viscoelastic fluid (0 < *a* < 1). For viscous fluids, the diffusion coefficient of the probe can then be used to determine the fluid viscosity. Alternatively, for viscoelastic fluids, the materials response is calculated from the creep compliance, *J*(*τ*), which is the time-dependent strain relative to the characteristic time of the material, namely the relaxation time, *τ*_*rel*_.

3 $$\langle J(\tau )\rangle =\,\frac{3\pi {r}_{h}\langle {\rm{\Delta }}{r}^{2}(s)\rangle }{2{k}_{B}T}$$

The creep compliance can also be directly converted to the frequency dependent elastic and viscous moduli. The diffusion of particles within a medium can be characterized as a probability distribution, referred to as a van Hove correlation function (equivalent to the MSD, ${\rm{\Delta }}{r}^{2}(\tau )$) that is calculated at a specific time lag, *τ*. In a homogenous medium the particle displacement is expected to follow a Gaussian distribution, while a deviation of the van Hove function (*I*_2_(*τ*) > 0.05) from Gaussian behavior indicates sample heterogeneity that can be defined by the excess kurtosis, *I*_2_(*τ*), as:

4 $${I}_{2}(\tau )=\,\frac{\langle {\rm{\Delta }}{x}^{4}(\tau )\rangle }{3{\langle {\rm{\Delta }}{x}^{2}(\tau )\rangle }^{2}}-1$$

This is derived from the ratio of successive moments.

### Confocal laser scanning microscopy (CLSM)

Confocal fluorescent images of fluorescently-tagged soluble dietary fiber and flamingo stained mucin were obtained using a Zeiss 710 Confocal laser scanning microscope (CLSM) with a C-Apochromat 40x/1.20 W Korr M27. Sequential excitation at 488 nm and 514 nm was provided by an argon laser. Emission filters MBS 488/561/633 and MBS 458/514 were used for collecting green and red fluorescent images with images saved and analyzed using Zeiss® software.

### Statistical analysis

Mean and standard deviation (SD) were reported with their respective *p* value and standard error. A value of *p* < 0.05 was considered statistically significant. The precision of the mucoadhesion assay was determined by analyzing three standard solutions of different concentrations. All experiments were performed in triplicate over three separate days under the same analytical conditions.

### Data availability

The datasets generated during and/or analyzed during the current study are available from the corresponding author on reasonable request.
